# Integration of Bite Mark Microbiome Analysis with Forensic DNA Profiling: Advancements, Challenges, and Synergistic Approaches

**DOI:** 10.5041/RMMJ.10528

**Published:** 2024-07-30

**Authors:** Palash Arun Mehar, Lina Zamsingh Bhoyar, Archana Laxminarayan Mahakalkar

**Affiliations:** 1 Department of Forensic Medicine & Toxicology, All India Institute of Medical Sciences, Raipur, Chhattisgarh, India; 2 Department of Forensic Biology, Government Institute of Forensic Science, Civil Lines, Nagpur, Maharashtra, India

**Keywords:** Bite mark, DNA profiling, forensic science, microbiome, saliva

## Abstract

Bite mark analysis plays a pivotal role in forensic investigations, by helping to identify suspects and establish links between individuals and crime scenes. However, traditional bite mark methodologies face significant challenges due to issues with reliability and subjectivity. Recent advances in microbiome analysis, which involves identifying and characterizing the microbial communities found in bite marks, have led to the emergence of a promising tool for forensic investigations. The integration of microbiome analysis with conventional DNA profiling enables more accurate interpretation of bite mark evidence in forensic investigations. This review provides an in-depth look at the integration of bite mark microbiome analysis with forensic DNA profiling. It also addresses the challenges and strategies involved in microbiome-based bite mark analysis for forensic purposes.

## INTRODUCTION

Forensic science is a multidisciplinary field that uses multiple techniques to analyze physical evidence in criminal cases. One such technique, bite mark analysis, significantly aids forensic investigations due to its ability to help identify the biter by matching their dentition pattern to the bite mark. Bite marks, defined herein as injuries caused by human teeth, are often found on victims of crimes or on items found at crime scenes and have been pivotal in solving cases such as assaults, sexual offenses, homicides, and child abuse.^1^,^2^ They represent a tangible link between the assailant and the victim. Their use invokes Locard’s exchange principles, which posit that contact between two items results in an exchange of materials.^3^,^4^ In this context, the exchange occurs when a bite mark leaves traces of the assailant’s dental characteristics on the victim’s body. Another equally important technique is forensic deoxyribonucleic acid (DNA) profiling. It has revolutionized criminal investigations and offers unparalleled specificity and reliability in identifying individuals.^5^

However, both techniques have limitations. Bite mark analysis has challenges related to analysis subjectivity, sample degradation, and concerns over its scientific validity.^6^ Forensic DNA analysis, on the other hand, can suffer from sample degradation or limited DNA samples.^7^,^8^ In light of these challenges, there is a pressing need for innovative approaches to enhance bite mark analysis reliability and accuracy in forensic investigations. One such approach involves integrating bite mark microbiome analysis with DNA profiling. This approach harnesses the unique microbial signature left within bite marks, which complements the genetic information obtained through DNA analysis.

This review paper explores the potential of integrating bite mark microbiome analysis with DNA profiling in forensic investigations. It delves into the principles behind bite mark analysis, the significance of this technique, the strengths and limitations of both bite mark analysis and DNA profiling, and the need for more reliable methodologies. By combining these two forensic disciplines, we aim to enhance the accuracy and reliability of forensic investigations, ultimately contributing to the pursuit of justice.

## THE PRINCIPLES AND SIGNIFICANCE OF BITE MARK ANALYSIS

Bite mark analysis has been a longstanding and invaluable forensic tool for identifying perpetrators of crimes. Conventional methodologies involve physically comparing the bite mark to known examples of the suspect’s teeth. Accurate documentation of physical evidence from both the bite mark (whether it is on human skin or an object that has been bitten) and the reference sample (consisting of teeth study casts) is crucial. Meticulous recording of these data ensures the best conditions for accurate comparisons and drawing meaningful conclusions.^9^

Compared to conventional approaches, advanced bite mark analysis techniques provide exact measurement, analysis, and visualization facilitated by three-dimensional (3D) scanning, computer analysis, and reverse engineering. Three-dimensional scanning enables precise, distortion-free imaging of bite marks in soft materials such as cheese, chocolate, and human skin.^10^ Cone-beam computed tomography (CBCT) provides reliable and non-destructive bite mark measurement. In 2018, Ali et al. reported that bite mark CBCT imaging was distortion-free and enabled accurate 3D measurements. Forensic bite mark analysis may also benefit from reverse engineering techniques.^11^ In 2023, Macorano et al. used software to patch together multiple photographs and then applied a reverse engineering technique to make digital 3D models of a bitten bun and the resulting dental models.^12^ However, larger sample numbers and more validation research are required.^11^

Traditional bite mark analysis has been criticized for being subjective and lacking in scientific rigor, particularly when the bite mark evidence is of poor quality or physical comparison is not possible.^13^ The elastic and distortable nature of skin, as well as the absence of a reliable impression medium, presents many challenges to bite mark analysis. In some cases, bite marks can cause lesions to cartilaginous structures (e.g. the ear), and the skin also retains the detected bite mark for a certain period. However, another aspect of bite marks is playing a significant role in forensic medicine: the saliva deposited during biting incidents.^9^

Bite marks are usually inflicted during violent acts such as sexual assault and murder, resulting in the transfer of the perpetrator’s saliva onto the victim. Consequently, saliva is frequently encountered in bite mark evidence during criminal investigations including homicide and assault. Analysis of this saliva, whether serological or cellular, plays a crucial role in identifying the perpetrator. While the presence of saliva alone may not conclusively prove that a crime was committed, it can establish a connection between individuals and a crime scene, serving as evidence of physical contact. When an adequate quality and quantity of saliva is collected at the crime scene, it becomes a valuable source of DNA. Consequently, detecting saliva contributes significantly to forensic investigations.^14^,^15^

## THE PRINCIPLES AND SIGNIFICANCE OF DNA PROFILING

Deoxyribonucleic acid (DNA) profiling plays a critical role in bite mark casework since it can provide indisputable evidence that identifies or excludes suspects based on distinctive genetic markers. In bite mark analysis, where biological substances such as saliva are often left behind, DNA profiling plays a pivotal role in establishing a direct connection between the biter and the bite mark, thereby aiding in identifying the perpetrator. Hence, DNA profiling is an invaluable tool that enhances the accuracy and reliability of investigations and contributes to the serving of justice with a high degree of certainty.

Forensic DNA profiling, also known as DNA fingerprinting, is a crucial technique in criminal investigations, particularly those involving bite marks. This method leverages each person’s unique DNA, preserved across various bodily tissues, enabling accurate discrimination between individuals.^8^

The DNA profiling process involves sample collection, DNA extraction, and amplification of specific DNA regions using techniques such as polymerase chain reaction (PCR). The resulting DNA fragments are then analyzed using methods such as gel electrophoresis or capillary electrophoresis. The resulting DNA profile consists of distinct alleles at specific loci, i.e. it is a genetic “fingerprint” that identifies or excludes individuals from suspicion based on matches or mismatches with reference samples.^8^

In bite mark analysis, the DNA is extracted from biological material found in the bite mark or from saliva left by the biter. The accuracy and reliability of DNA profiling have revolutionized forensic investigations, significantly improved the precision of individual identification, and assisted in securing both convictions and exonerations.^8^

Significant technological advancements in DNA profiling have been made since its development in the 1980s, enabling the utilization of human biological sources for identification purposes. When dealing with cases involving bite marks, the human DNA retrieved from saliva has been proven to be a dependable form of evidence.^16^ Nonetheless, the presence of relatively high concentrations of nucleases found in saliva, like deoxyribonuclease I, leads to the rapid degradation of exposed DNA. Consequently, obtaining the quality and quantity of salivary DNA needed to generate a DNA profile can be a formidable task.^17^–^20^ Hence, an alternative approach to bite mark analysis has emerged, which focuses on bacterial genotyping methodologies—microbiome analysis.^21^–^22^

## FILLING THE GAP: BITE MARK MICROBIOME ANALYSIS

Microbiome analysis involves study of the bacterial community in a given environment. Bacterial DNA is shielded by a cell wall, which acts as a natural barrier against the degradation experienced by exposed human DNA. Due to the diversity and ubiquity of microbial DNA, its increased resistance to degradation (attributable to the cell wall and bio-film), and the potential for differentiating between monozygotic twins, microbiome analysis can offer substantial advantages over DNA analysis alone.^23^,^24^

Hence, bite mark microbiome analysis is emerging as a promising forensic tool. This innovative technology offers an unbiased and dependable approach to identifying individuals involved in criminal activities by examining the microbial communities present within bite marks.

### The Salivary Signature and Microbiome Bite Mark Analysis

A salivary signature contains information regarding an individual’s age, gender, personal traits, and health. It is created from the salivary traces found in bite marks.

The salivary microbiome consists of bacterial genomes present at a particular location. In humans, microbiome generally refers to all distinct microbiota found throughout the human body.^25^ For instance, saliva is estimated to contain 700 distinct bacterial species, with a concentration of approximately 1.4×10^8^ microorganisms per milliliter.^26^ Numerous bacteria, such as *Actinomyces, Campylobacter, Capnocytophaga, Corynebacterium, Fusobacterium, Haemophilus, Lactobacillus, Neisseria, Prevotella, spirochetes, Streptococcus*, and *Veillonella*, are frequently present in saliva.^25^

When studying the salivary microbiome, exams are performed to ascertain the proportion of different bacteria found in the saliva; this information may aid in subject identification.^27^ A variety of factors determine the composition of the salivary microbiome, including age, circadian rhythms, lifestyle choices, dietary habits, cohabitation status, smoking habits, interpersonal interactions such as kissing, and overall state of health.^23^,^28^,^29^ However, more research is needed to establish a robust link between bite mark microbiology and specific behavioral traits. While the analysis of salivary signatures and the microbiome holds promise for forensic investigations, significant challenges remain. A deeper understanding of the factors influencing microbial stability and the establishment of standardized guidelines for sample collection and analysis are essential.

### Microbiome Stability and Time Since Infliction

Bite mark microbiome analysis and time since infliction provide important insights into the microorganisms present in human bite marks and determination of the period between bite infliction and examination. These analyses provide invaluable information about suspect identification, victim healing progress, and the timeline of events. By analyzing microbial communities within the bite mark, researchers can identify unique bacterial species associated with oral cavities, aiding in suspect profiling. Additionally, examining change in bacterial composition over time enables estimating the time passed since a specific bite occurred. Factors such as lesion age, host response mechanisms, and environmental conditions influence microbial colonization and subsequent growth within the wound site. Variations in these factors may impact microbial growth and survival in bite mark samples.^30^ Therefore, understanding bite mark microbiota dynamics along with an accurate assessment of the elapsed time since infliction enhances forensic investigation accuracy and enables more comprehensive analyses of crime scenes.

Borgula et al. provided compelling evidence that bacteria clearly originating from the oral cavity can be successfully retrieved from bite marks left on human skin for up to 24 hours. This bacterial genotyping approach can potentially contribute to perpetrator identification since the bacteria recovered from bite marks can be exclusively matched to the suspects’ teeth in the samples acquired (usually 8) due to the considerable genetic diversity among oral streptococci.^21^

*Streptococcus salivarius* is predominant in saliva, and 45–50% of bacteria are lost from the infection site every hour.^31^ Hence, the amount of bacteria that can be recovered decreases over time. Brown et al. found that prominent amplicons could be found no more than 48 hours after bite mark infliction. The authors concluded that although bacterial samples recovered from dead bodies are more likely to remain viable for longer, in living people they may become less viable over time due to bathing, washing, or antiseptic applications to the bite site.^31^

These studies provide insights into the temporal dynamics of the oral microbiome. However, research is ongoing, and further validation is needed to establish robust methodologies for estimating the post-infliction interval based on microbial data. Nevertheless, the potential for microbial succession patterns offers a promising avenue for forensic investigations, enhancing our ability to determine the timing of bite mark inflictions.

## THE STAGES OF DNA PROFILING AND MICROBIOME ANALYSIS

**Figure 1:**
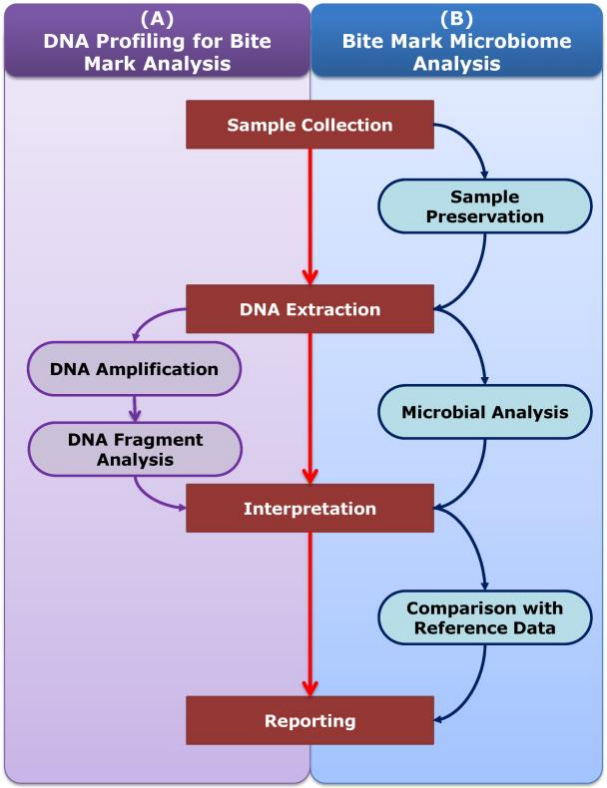
Different Stages of DNA Profiling and Microbiome Analysis in Bite Mark Examination.

Figure 1 illustrates the different stages of DNA profiling and microbiome analysis in bite mark examination. Note the clear sequential steps involved in DNA profiling and studying microbial communities for enhanced forensic investigations. Sample collection, DNA extraction, and sequencing techniques provide a fascinating avenue for forensic investigations by leveraging the world of microbiome analysis within bite marks. As the microbiome leaves its microbial mark, it offers valuable information to assist in the investigation of bite wounds.

### Saliva Collection

Detectable salivary remnants can be retrieved as evidence for the purpose of identity testing. Ensuring the preservation and integrity of the micro-biome in the collected saliva is crucial and requires a careful and meticulous approach. To capture the unique composition and profile of microbial communities within the sample, forensic experts use either the single-swab or dual-swab technique. The single-swab method involves using a moistened cotton swab to collect saliva from the skin immediately after an injury to prevent DNA degradation. The dual-swab method uses a moistened swab followed by a dry swab, both rolled across the skin to maximize evidence collection, and then air-dried for at least 30 minutes.^12^,^32^

### DNA Extraction

After collecting the saliva from the bite mark, the microbial DNA must be extracted for analysis. Accurate and reliable results depend on the quality of the genetic material obtained. Hence, an effective and fast DNA extraction process is of utmost importance. Bite mark analysis commonly employs a range of DNA extraction techniques, including phenol-chloroform extraction, commercial DNA extraction kits, and more advanced enzymatic extraction techniques.^12^

### DNA Sequencing

After extracting the microbial DNA, sequencing techniques are used to reveal the microbiome residing within bite marks. The advent of next-generation sequencing methods has led to marked progress in the field of microbiome analysis. Techniques such as amplicon sequencing and shotgun metagenomics are frequently utilized for studying microbiomes in bite marks. These techniques enable the identification and characterization of the microbial DNA present in the samples. Amplicon sequencing targets specific regions of the microbial genome, providing information on microbial diversity, while shotgun metagenomics allows for a more comprehensive analysis of the entire microbial genome.^33^

To achieve a focused analysis of bacterial populations, 16S rRNA gene sequencing targets a conserved bacterial gene region. Though not as exhaustive as shotgun sequencing, this method, noted by Caporaso et al. in 2011, offers insights into the relative abundance and diversity of bacterial taxa and has proved popular for its cost-effectiveness and simplicity.^34^

Various high-throughput sequencing (HTS) methods have transformed DNA analysis, notably with regard to bite mark analysis in forensic investigations. Prominent HTS systems such as MiSeq (Illumina, San Diego, CA, USA) and Ion Torrent (Thermo Fisher Scientific, Inc., Waltham, MA, USA) facilitate swift and simultaneous sequencing of millions of DNA fragments, providing an extensive view of microbial DNA within a given specimen. This approach, described by Caporaso et al. in 2010, yields insights into microbial diversity, abundance, and functional potential.^35^

Combining multiplex PCR with HTS offers a targeted approach to amplify multiple DNA regions in a single reaction. Researchers can design specific primers for microbial and human targets, allowing simultaneous profiling of both DNA types from bite marks. This approach, detailed by Sinha et al. in 2015, provides a comprehensive genetic profile in a single assay, facilitating forensic investigations.^36^

Nanopore sequencing, a portable and real-time DNA sequencing technology, stands out for its ability to concurrently analyze microbial and human DNA. This technique, described by Quick et al. in 2014, involves threading DNA strands through nanopores and generating electrical signals corresponding to DNA bases. It enables rapid sequencing of complex mixtures and holds potential for on-site forensic analysis.^37^

Single-cell sequencing is a breakthrough approach that allows the study of individual microbial cells without the need for cultivation. This technique, detailed by Quince et al. in 2017, reveals genomic diversity within microbial communities and aids in the identification of rare or unculturable species.^38^

When choosing a sequencing technique, considerations such as cost, throughput, and the specific research question at hand come into play. Each technology has its own strengths and limitations, and it is important to select the most suitable approach for the specific forensic analysis being conducted.

## RESEARCH METHODOLOGIES FOR BITE MARK MICROBIAL ASSESSMENT

A brief overview of the various methodologies used to analyze the microbiome of human bite marks underscores the microbiome's importance for the integrity of forensic investigations.

### Streptococcal Diversity and Initial Analysis Techniques

*Streptococci*, found in the oral cavities of most individuals, exhibit remarkable genetic diversity. Research has identified significant quantities of *Streptococcus* in bite marks on human skin, which can correlate with samples taken from the biter’s teeth. Studies using the arbitrarily primed polymerase chain reaction (AP-PCR) technique, which enables rapid analyses of a larger number of oral bacteria, have further supported these findings. This innovative method shows promise for bite mark analysis, particularly when the biter’s DNA cannot be retrieved.^39^

Back in 1984, Elliot et al. investigated the feasibility of using pyrolysis mass spectrometry to differentiate between isolated *Streptococcus salivarius* saliva samples obtained from two different individuals. The researchers concluded that *S. salivarius* sample analysis was capable of differentiating between two distinct individuals.^40^

In 2005, Rahimi et al. investigated the effectiveness of AP-PCR in tasks such as biter identification, assessing the natural distribution of oral *Streptococcus* genotypes, and examining their recoverability after a 12-month period. Their results indicated that AP-PCR enables rapid analysis of a significant number of bacteria while maintaining high resolution, thus showcasing its potential for use in forensic investigations.^6^

### Advancements in Bacterial DNA Analysis Techniques

In 2012, Kennedy et al. studied the feasibility of matching bacterial DNA sequences extracted from experimental bite marks with those extracted from the corresponding teeth. They evaluated the discriminative capacity of three specific genomic regions within streptococcal DNA to differentiate between samples originating from different individuals. Their study provided compelling evidence that amplified bacterial DNA from both bite marks and teeth can offer valuable corroborative data for perpetrator identification.^13^

Also, in 2012, Hsu et al.’s research assessed the reliability of directly amplifying bacterial DNA extracted from bite marks and comparing it to DNA from oral samples. The researchers used PCR with primers specifically designed for amplifying the streptococcal 16S rDNA. The resulting amplicon profiles were then compared using denaturing gradient gel electrophoresis (DGGE). Notably, when analyzing bite marks with 6 or more DNA bands, 8 out of 15 coincided with the corresponding incisor. Hence, they found that direct amplification of streptococcal DNA extracted from a bite mark provides valuable data for bite mark identification and comparison.^22^

### Protein Analysis and Microbial Identification

The distinct protein composition of the microbiome in bite marks is referred to as the “protein signature.” The protein signature can help identify certain bacterial species often present in the oral microbeome, such as *Fusobacterium, Haemophilus, Neisseria, Porphyromonas, Prevotella, Rothia, Streptococcus*, and *Veillonella*. Examination of these proteins in the bite mark microbiome may help identify certain subjects in forensic investigations.^27^,^41^

Microarrays aid in microbial identification, characterization, and community analysis of microbial communities in bite marks. Certain microbial species and diseases can be identified and characterized using PCR to amplify target sequences, followed by hybridization onto microarrays. Functional gene arrays (FGA) and community genome arrays (CGA) enable simultaneous investigation of several microbial communities in bite marks, shedding light on population dynamics and environmental effects. Furthermore, microarrays with probes targeting repetitive sequences can distinguish bacterial species and help assess microbial contamination. Microarray technology contributes to clinical diagnoses, forensic investigations, and the understanding of microbial interactions in bite wounds.^42^

### High-resolution and Genomic Techniques

Several studies have employed different techniques to analyze *Streptococcus* genotyping. These approaches encompass gene amplification techniques (e.g. targeting the 16S rRNA gene) and direct pyrosequencing. Additionally, techniques such as DGGE of the 16S rRNA, AP-PCR, and whole genomic “fingerprinting” using various restriction endonucleases have been employed.^6^,^13^,^21^,^22^ In forensic scenarios, the bacterial levels in most samples are relatively low, necessitating the use of highly sensitive methods. Therefore, standardized laboratory protocols for sample collection and analysis must be used, along with bioinformatics tools for data analysis. Doing so helps mitigate biases arising from different extraction protocols, PCR reactions, and sequencing platforms, facilitates data integration, and fosters seamless communication among researchers.^43^

### Microbiome-based Bite Mark Comparison

In forensic analyses, scientists compare microbiome-based bite marks by examining bacterial DNA sequences extracted from both the bite mark and the suspect’s teeth to confirm a match.

This type of comparison is a valuable supplementary tool when traditional morphometric analyses, which rely on shape and size, fail to provide conclusive or reliable results. The unique diversity of the oral microbiome can differentiate between individuals—a microbial fingerprint that establishes or excludes a match, thereby enhancing the accuracy and reliability of forensic investigations. This approach complements existing methods and provides a more comprehensive and robust analysis, ultimately aiding in the pursuit of justice.^10^,^44^

### Limitations of Microbiome-based Bite Mark Comparison

This method does have some limitations. Firstly, it requires high-quality and high-throughput sequencing technologies, which can be costly or even unavailable in certain settings. However, alternative sequencing technologies offer a balance between cost and quality, such as targeted amplicon sequencing or metagenomic shotgun sequencing. These methods can provide valuable microbial information more cost-effectively than traditional HTS.

High-throughput sequencing is susceptible to contamination, degradation, or inadvertent bacterial DNA transfer from other sources, which potentially compromises the accuracy and reliability of the results. Hence, strict contamination control protocols should be implemented during sample collection, processing, and analysis. This includes using sterile techniques, negative controls, and results validation to ensure that any microbial DNA detected is indeed from the bite mark and not from external sources.

Finally, the dynamic nature of the oral microbiome over time or under differing circumstances may not be fully considered when using HTS. Hence, this methodology might not provide a comprehensive understanding of the behavior and changes in the oral microbiome. However, this limitation can be addressed by conducting longitudinal studies to capture the variability and dynamics of the oral microbiome over time and under various conditions. This will help in understanding how the oral microbiome responds to different factors and changes, offering a more thorough perspective for bite mark analysis.^10^,^33^

## THE COMPLEMENTARY NATURE OF BITE MARK MICROBIOME ANALYSIS AND DNA PROFILING

Researchers constantly strive to develop new and improved methods of forensic investigation. Integrating innovative methodologies makes it possible to amplify the strengths inherent to each method and achieve synergistic benefits. The combination of bite mark microbiome analysis and DNA profiling represents one particularly powerful synergy. This novel integration merges the unique microbial signatures found in bite marks with the genetic individuality encoded in DNA, offering a comprehensive and robust approach to forensic identifications.

The integration of bite mark microbiome analysis with DNA profiling techniques has greatly improved forensic investigations. This collaborative approach not only strengthens the evidentiary value but also overcomes the limitations associated with traditional DNA profiling (Table 1). Hence, microbiome analysis offers a holistic perspective of an individual that complements the genetic information obtained from DNA analysis alone. By examining the microbial composition within bite marks, investigators can uncover valuable insights into an individual’s distinct characteristics, dietary habits, lifestyle choices, and interactions with their surroundings. This supplementary information greatly enhances forensic investigations, potentially leading to more accurate identification and a deeper understanding of the individuals involved.

**Table 1:** Information Gleaned when Integrating Bite Mark Microbiome Analysis with DNA Profiling.

Benefit	Information Gained	Contribution to Forensic Investigations
Enhanced identification accuracy	Unique microbial signature for each individual^8^,^45^ Includes information on the microorganisms within the human body, encompassing both the internal and external environment^46^	Enhanced accuracy in validating perpetrator identity and linking individuals to crime scenes
Complementary evidence	Microbial DNA can be detected even when human DNA is limited, degraded, or contaminated^47^	Enables access to a broader range of evidence samples and reduces the number of false positive identifications
Extension of post-bite mark interval for analysis	Microbial DNA remains intact after significant time periods,^47^ compared with traditional DNA that degrades over time	Useful for cold case investigations
Holistic crime scene reconstruction	Provides information about the environment, diet, possible social interactions, tobacco use, alcohol consumption, health conditions, and more^45^ Microbial communities are influenced by social interactions and a person’s daily routines^46^	Contributes to suspect profiling and the crime scene context Detecting potential connections between suspects or witnesses
Human–microbe interaction dynamics	Provides information on the intricate relationship between human hosts and the microorganisms residing within Environmental microorganisms can be transferred during a biting incident	Contributes to investigation of factors influencing a biting incident and contributes to the reconstruction of surrounding events^45^
Geographical/temporal clues	Microbiome data reflect known specific microbial differences in geography and the environment^45^,^48^ Microbial communities demonstrate temporal stability over shorter time frames^40^ and rapidly adapt in response to environmental fluctuations^45^	Geographical region can be deduced from microbiome data based on known specific microbial differences in geography and environment^45^,^48^ and give insights into geographic locations visited by a suspect

## STANDARDIZATION AND VALIDATION IN FORENSIC MICROBIOLOGY

While there are some established procedures and kits for forensic microbiological analysis, the field is still evolving, and standardization varies across different contexts and jurisdictions.

In 2014, Budowle et al. worked on microbiological forensic applications and validation of HTS. They underlined the necessity of meticulous validation in creating and using microbiological forensic techniques, stressing the significance of verifying the usefulness of HTS under specified operating parameters and limitations. To guarantee the validity and dependability of microbiological forensic studies and, eventually, improve public safety and national security (e.g. microbial evidence that could be linked to bioterrorism, disease outbreaks, etc.), the authors provided guidelines for HTS system validation that addressed sample preparation, sequencing, and data processing.^49^

Budowle and colleagues also brought to light the difficulties and limitations of microbial forensics, and offered insightful information for validation strategies and procedures for various process phases.^49^ To guarantee dependability and credibility in forensic investigations, they also discussed the need for appropriate validation in gathering, preserving, transporting, analyzing, interpreting, and communicating probative evidence.

A 2019 study by Alessandrini et al. focused on a general DNA extraction technique for microbial and human DNA analysis from a single trace. They demonstrated that the DNA IQ^TM^ Casework Pro Kit for Maxwell® (Promega, Madison, WI, USA) can extract DNA from different types of samples, which is important for forensic laboratories analyzing limited samples of human DNA.^50^

### Addressing Validation and Standardization Challenges

Validating and standardizing microbiome-based bite mark analysis techniques presents significant challenges that must be addressed to ensure the reliability and acceptance of this innovative approach in forensic investigations.

Table 2 presents a comprehensive overview of the challenges and corresponding strategies linked to microbiome-based bite mark analysis. Effectively addressing the challenges related to sample collection, database management, workflow integration, legal admissibility, and ethical considerations is essential. Only by doing so can the advantages offered by microbiome-based bite mark analysis be fully exploited.

**Table 2:** Challenges and Strategies Associated with Microbiome-based Bite Mark Analysis.^33^,^46^,^47^,^51^,^52^

Challenges	Strategies
Method standardization	Develop consistent guidelines for collecting, handling, and preserving samples. Specify standardized methods for DNA extraction, sequencing, and analysis. Standardize metadata collection along with microbial data, including sample details, collection protocols, and environmental factors, to enhance data interpretation.
Validation studies	Curate a reference database of oral microbiomes from diverse populations, age groups, and oral health conditions. Integrate metadata (e.g. geographic location, lifestyle) to contextualize the microbial profiles.
Quality control and assurance	Implement strict quality control steps to identify and correct technical artifacts, contamination, and batch effects during DNA extraction, sequencing, and analysis. Regular monitoring and validation of laboratory procedures are necessary to ensure accurate results.
Data interpretation and integration	Develop algorithms and software tools for integrating microbial DNA and human DNA profiles to establish reliable associations. Establish criteria for determining the strength of the microbial evidence and its correlation with human DNA evidence.
Microbial database development and curation	Regularly update the reference database to include new microbial species and variations discovered through ongoing research. Implement protocols for maintaining data integrity, ensuring accessibility, and safeguarding against data loss.
Microbial biomarker identification	Identify specific microbial biomarkers associated with individuals, which can enhance the discriminatory power of the analysis.
Machine learning integration	Integrate machine learning tools to aid in taxonomic classification and identify discriminatory microbial signatures associated with individuals.
Sample collection and preservation	Investigate and optimize methods for preserving microbial DNA in bite mark samples over time for accurate analysis.
Population diversity consideration	Account for population-specific variations in oral microbiomes by including diverse population samples in the reference database.
Case studies and real-world testing	Apply microbiome-based analysis to real forensic cases to assess its practical applicability, reliability, and effectiveness.
Forensic reporting guidelines	Establish clear guidelines for reporting microbiome-based evidence in forensic reports to ensure transparency and clarity.
Courtroom acceptance and communication	Work with legal experts to ensure that microbiome-based evidence meets the standards for admissibility in court proceedings. Communicate results effectively.
Ethical considerations and informed consent	Develop ethical guidelines for the collection, use, and sharing of microbiome data and ensure informed consent from participants.
Integrated analysis validation and accreditation	Conduct blind testing where laboratories analyze the same samples without knowledge of their origins to assess consistency. Collaborate with regulatory bodies to establish guidelines and accreditation standards for microbiome-based forensic analysis, ensuring adherence to quality and ethical standards.

## FUTURISTIC AVENUES IN MICROBIOME-DNA INTEGRATION FOR FORENSICS

The fusion of microbiome analysis with DNA profiling holds immense potential for future advancements in forensic investigations. Particularly promising is the establishment of integrated analysis platforms that markedly simplify the interpretation and reporting procedures.

For example, incorporating advanced imaging techniques such as 3D scanning or dental impressions with microbiome DNA analyses can augment the evidence and more accurately match bite marks to suspects.^10^ Furthermore, by integrating machine learning algorithms, new avenues would open for processing intricate multidimensional data, refining identification precision, and enabling effective pattern recognition.^10^,^53^

In addition, the incorporation of novel forensic techniques, such as chemical analysis of bite marks or advanced imaging, could greatly enhance the forensic toolkit. These techniques would offer a more expansive perspective on evidence and strengthen the validity of findings.^53^

Modern advancements in the forensic sciences necessitate the fostering of interdisciplinary collaboration among microbiologists, geneticists, statisticians, and legal professionals. This is essential for establishing standardized protocols, refining methodologies, and ultimately improving the overall reliability of integrated approaches.^54^

## CONCLUSION

The integration of bite mark microbiome analysis with forensic DNA profiling presents a promising avenue for advancing the field of forensic science. This innovative approach has the potential to revolutionize the way bite mark evidence is analyzed and interpreted, leading to more accurate and reliable results. The advancements made in this area have shed light on the intricate relationship between the human microbiome and bite mark evidence. Forensic investigators can gather valuable information about the perpetrator’s identity, lifestyle, and even geographical origin by analyzing the microbial communities present in bite marks. This additional layer of evidence can significantly enhance the probative value of bite mark analysis, providing a more comprehensive and robust investigative tool. With continued advancements, standardized protocols, and interdisciplinary collaboration, this approach has the potential to revolutionize bite mark analysis, providing more accurate and reliable evidence in criminal investigations.

## Abbreviations

16S rRNA: 16S ribosomal-ribonucleic acid
3D: three-dimensional
AP-PCR: arbitrarily primed polymerase chain reaction
CBCT: cone beam computed tomography
CGA: community genome arrays
DGGE: denaturing gradient gel electrophoresis
DNA: deoxyribonucleic acid
FGA: functional genome array
HTS: high-throughput sequencing
PCR: polymerase chain reaction
